# Hypnotism

**Published:** 1891-08-29

**Authors:** 


### HYPNOTISM.

Few intelligent people would now require to be told what is meant by hypnotism. To the general public it is but another term for mesmerism; for although the term was invented about forty years ago by James Braid, surgeon, of Manchester, for the system which he built up, and which he based on the psychical phenomena known at that time as mesmerism, or animal magnetism, the term has now been almost universally adopted for the degrading public exhibitions which have recently become only too common.

Many of the phenomena of hypnotism have been observed by professional mesmerists and magnetisers from a very remote period. In the writings of Paracelsus we have the earliest evidence of a belief in the existence of magnetic properties in the human body. Later in the sixteenth century a tradition arose that "ascribed to man the power of exercising in his fellows an action analogous to that of the magnet," whence originated the term animal magnetism. Anthony Mesmer was a German, who lived in the latter half of the eighteenth century, and attracted a great deal of attention by his crude hypnotic practices in Paris, but met with such an amount of professional opposition that he had to leave France.

For those who desire further historical information on the subject, a good account will be found in Binet and Féré's book on "Animal Magnetism."

In 1841, Braid having observed the practices of Lafontaine, a former pupil of Mesmer, investigated the subject, and explained rationally and scientifically many of the phenomena. In 1846 Esdaile began in India to operate on patients under hypnosis, and the fact that no pain was felt during the operations excited much wonder. However, the whole subject seems to have been dropped by the medical profession and relegated to charlatans and quacks, till it was revived by Dr. Liébeault, of Nancy, who began, about thirty years ago, to practice hypnotism in the public dispensary at Nancy. He soon hit upon the essential point upon which the value of hypnotism depends, viz., suggestion in hypnosis.

As Liébeault must be regarded as the father of modern hypnotic therapeutics, we cannot do better than refer to his own writings for what he considers the best method of obtaining this hypnotic sleep. "Having caused my patient to recline in an arm-chair, or, better still, upon a sofa, I bid him fix his eyes upon mine. While his mind is concentrated upon obeying me, while his other senses are becoming dulled and impercipient of impressions from without, I recapitulate to him the preliminary phenomena of sleep—torpor of the limbs, drowsiness, heaviness of the eyelids, &c.—and when I notice that the eyelids quiver and droop, or that the eyes assume a surprised and dazed expression, I give the word of command—"Sleep!" If, after that injunction, the eyes still remain open, I repeat my list of affirmations, and, finally, close my patient's eyelids. In the cases of workmen, peasants, children, and soldiers, who all are accustomed to obey, the eyes generally close mechanically when I say the word "Sleep!" There is nothing new under the sun. I adopt a method which has long been known to magnetisers, and I add to it verbal suggestion, employed long ago by Faria to induce sleep. But I add something more; I take into account our proneness to imitate the actions of our surroundings, and the ease with which we are borne into slumber when our minds are reassured and calm. Therefore, I hypnotise each of my patients in the presence of fifteen to twenty others, so that while each awaits his turn he may become familiarised with my method, and interested in the results which he is allowed to witness. He is thus led to forget himself, and falls under an imitative influence which leads him, when his turn has come, to pass easily into the same state which he has watched his neighbours entering." Further, Liébeault states that the theory of the production of sleep being once understood, it is easy to understand how the awaking is accomplished. As the state is brought about by suggestion, so by suggestion it is dispelled. It generally suffices to bid the patient awake, but he is often helped to obedience by the waving of a fan or screen in front of his face. This indirect suggestion, and the sensation of coolness, add their influence to the verbal command, and the patient emerges from the hypnotic state, even more readily than he entered it. This method, he says, is not only the surest and most speedy, but also the most scientific; and it almost invariably succeeds when the hypnotiser has confidence in himself, and a proper amount of experience. In this way Liébeault has succeeded in hypnotising over nine-tenths of his patients.

It is impossible here to detail the various methods by which hypnosis may be induced. In methods such as that of Liébeault, it is believed that the prolonged staring in a fixed manner at a bright object in a strained attitude results in fatigue of the retina and ocular muscles, and this prolonged stimulation, resulting in exhaustion, extends through their visual and oculomotor nerve centres to other sensory and motor nerve centres in the brain, and that thus a peculiar condition of the brain cells is induced which is somewhat analogous to the condition of these nerve centres in ordinary sleep. The brain is, as a rule, not capable of spontaneously originating any movement, whilst all its functions may be influenced by suggestions from without.

Professor Charcot has shown that the hypnotism represents clinically a condition comprising a series of nervous states. Now, according to Charcot these different states may be referred to three fundamental types: (1) The cataleptic state; (2) the lethargic state; (3) the state of induced somnambulism. It is now almost universally held that these three types do not represent any natural stages in hypnosis—they may be cultivated in any one, but do not occur without suggestion.

In the first two of these conditions the patient is quite conscious, but is drowsy and lethargic, and has lost voluntary control of his muscles. He is able to hear and answer questions put to him by anybody, and is not only *en rapport* with the hypnotiser. In the third stage, *Somnambulism*, "Le Grand Hypnotisme" of Charcot, the patient is quite unconscious, and appears to be sound asleep. He is deaf to all but the operator, but is absolutely in his power. Not only is the somnambulistic subject bound to perform any act that the operator suggests, but even his thoughts are at the mercy of the operator. Any delusion may be foisted upon him; he may be told to sign his name to any document, and he has no choice in the matter. But what is a far more serious matter, he may be compelled to do anything, or even to think anything that the operator may suggest he shall do or think after he has been restored to his waking condition, and this "*post-hypnotic suggestion*" in many cases certainly holds good for a considerable period of time; and in carrying out this post-hypnotic suggestion he may be absolutely unconscious of the origin of the impulse, which may be irresistible. Dr. Lloydd Tuckey reminds us that this is the condition to which the unfortunate victims of professional magnetizers are reduced. The greatest danger to the public lies in post-hypnotic suggestion, for it is almost certain that a weak mind may commit a crime as the result of such a suggestion. Our readers will recollect that it was recently urged in Gabriel Bompard's defence that she committed the Gouffe murder under the influence of such a suggestion.

Now it is claimed that this post-hypnotic suggestion may be utilised in therapeutics, and we may illustrate its practical application by the following case of deafness in both ears which was cured in one séance, reported by Dr. Burot, of Rochefort. A girl, aged 19, became suddenly unconscious from grief at parting from a relative. She remained unconscious for an hour, and upon recovering consciousness she was almost completely deaf, not being able to hear anyone who spoke to her. She was delirious for several nights following, and was distressed with the idea that she had lost the power of hearing. It was the opinion of her medical attendant that she would never recover it. Six days after the first onset, the patient was hypnotised by Burot. In about ten minutes a slight tremor of the limbs occurred, and Burot said "On recovering, Mademoiselle, you will hear the name Elise when it is pronounced." About five minutes later she was awakened, and said "I hear!" She not only heard the word Elise, but all that was said to her. It had been intended to fix the suggestion upon one word only, as advised by Bembeim and Bérillon. The patient stated that the first word she heard had been "Elise," but probably without noting the fact, she completed the suggestion by saying to herself "Since I hear the name 'Elise,' I should be able to hear all words." The patient is stated to have been completely cured.

In 1889 Voisin opened a discussion in the psychology section of the British Medical Association on the utility of hypnotism in the treatment of insanity. He affirmed that he had not found that the insane were incapable of hypnosis, as was generally supposed, and that by this method he had cured persons suffering from hallucinations and delusions, and from disturbances of special and general sensation. Suicidal ideas and acute mania had disappeared by its use. He also claimed similar success in dipsomania, morphinomania, and other affections. In the discussion which followed his statements were supported by various physicians who had seen the same results at the Salpêtrière: but were adversely criticised by others who had tried the method in English asylums, but generally without success. The Committee on Hypnotism appointed by the Association last year have asked that further time be allowed them for completing their researches. They, however, expressed a most emphatic opinion as to the dangers which its use by unqualified persons might lead to.

But hypnotism is by no means the innocent agent as it is represented. The mind may undoubtedly be impaired by a frequent induction of somnambulism, and the subject may be rendered almost idiotic. We entirely agree with Mr. Ernest Hart when he declares that he is led to believe that hypnotism is a proceeding directly dangerous, because it may end in completely upsetting the intelligence of the subject, and indirectly by the excessive influence which it gives to the operator over the subject, of which the limits have not yet been determined. Public exhibitions of hypnotism have been made illegal in France and over the greater part of the European continent and in many American States, and it is high time that they were forbidden in England. We point the finger of scorn at the Spaniard for revelling in his bull fights, but we go in crowds to amuse ourselves with watching the ridiculous attitudes and acts of hypnotised human beings, whose will and intelligence are for the time being completely suspended. Heaven forbid that science should connive at these vulgar shows of hypnotism, which serve no useful purpose and are often fraught with danger.

Many a medical man could tell of alarming conditions resulting from improper attempts at hypnotising by the unskilled amateur. One such case has quite recently been reported by Dr. Solon. An amateur at a friend's house volunteered to hypnotise another visitor and after two trials succeeded so well that the subject became extremely excited, lost the power of speech, and then passed into the condition of catalepsy; subsequently he had severe convulsions. He had simply been hypnotised by being made to look at a diamond ring, and afterwards the sight of anything glistening threw him into a state of violent excitement. The floor of the room in which the physician discovered him was covered with cushions as he frequently threw himself from the sofa on to the floor and was in a condition of grave hysteria with maniacal excitement. He was treated with full doses of sedatives, chloral, sulphonal, bromides, and morphine, but at first showed no improvement. After ten days the convulsive attacks were replaced by periods during which he sang persistently; he would sing every song he knew without stopping. After a fortnight of this he had a high temperature for several days, and altogether was very ill for three weeks. Such cases are not so uncommon as is generally supposed.

It is fairly certain that hypnotism may be employed with advantage as a therapeutic agent in certain nervous affections, but that if it is a potent agent for good, it may equally be a grave source of danger, and that we cannot inhibit the highest centres of the cortex and make the suspension of consciousness and intellect a source of amusement with impunity. It should be only permissible to hypnotise for therapeutical purposes, and then we should observe the golden rules laid down by Beaunis: Never to hypnotise except with the patient's free consent, and, if necessary, that of the friends; (2) never to make any experiment without the knowledge and consent of the patient; (3) never to operate except in the presence of a third person.

With these precautions the skilled hypnotist can do much to relieve and cure many conditions that are scarcely amenable to any other known therapeutical agent.

